# The global incidence rate of type 2 diabetes related chronic kidney disease and predictions by Bayesian age-period-cohort analysis: findings from the Global Burden of Disease Study 2019

**DOI:** 10.3389/fendo.2025.1429048

**Published:** 2025-08-11

**Authors:** Junpu Yu, Fanhui Luo, Yiwen Zhang, Jingli Yang, Shuxia Yu, Nan Li, Aimin Yang, Li Ma, Jinsheng Li

**Affiliations:** ^1^ School of Public Health, Lanzhou University, Lanzhou, Gansu, China; ^2^ Department of Medical Administration, Gansu Provincial Maternity and Child-care Hospital, Lanzhou, Gansu, China; ^3^ Department of Medicine and Therapeutics, The Chinese University of Hong Kong, Prince of Wales Hospital, Hong Kong, Hong Kong SAR, China

**Keywords:** Bayesian age-period-cohort model, chronic kidney disease, incidence, sociodemographic index, type 2 diabetes

## Abstract

**Aims:**

To evaluate the spatial-temporal changes in the incidence of type 2 diabetes related chronic kidney disease (CKD-T2DM) from 1990 to 2019, categorized by age and sex in 21 regions with different socio-demographic indexes (SDI), and to predict the incidence rate between 2020 and 2030.

**Methods:**

Data on the burden of CKD-T2DM were obtained from the Global Burden of Disease Study 2019. Age-standardized incidence rates (ASIR) were estimated by sex, age, region, SDI, and specifically in China. The trends of ASIR were assessed using Joinpoint model to calculate the average annual percentage changes (AAPCs) and their 95% confidence intervals. Prediction was conducted using the Bayesian age-period-cohort (BAPC) model.

**Result:**

In 2019, the ASIR of global CKD-T2DM increased with age in both sexes, and was highest in the older 75 age group. The ASIR of CKD-T2DM in males was higher than those in females. Overall, the global ASIR of CKD-T2DM increased from 1990 to 2019 in both sexes and all age groups. The most significant increase was observed in the 15–49 age group [males: AAPC=1.42, 95%CI:(1.35-1.49); females: AAPC=1.18,95%CI:(1.13-1.23)]. Besides, the upward trends in ASIR of CKD-T2DM were observed in most SDI regions and GBD regions. The changing trends in ASIR of CKD-T2DM in China were similar to the global trends. Finally, the predicted ASIR was also found to be increased globally and also in China in both sex from 2020 to 2030.

**Conclusion:**

The global CKD-T2DM incidence rates increased from 1990 to 2019 in both sexes, most regions and in China., and also increased globally between 2020 and 2030. Therefore, it is important to input more medical resources and establish prevention strategies for the increasing trends of CKD-T2DM.

## 1 Introduction

Chronic kidney disease (CKD) is a non-communicable disease with high incidence and mortality rate, typically diagnosed by persistent albuminuria and/or persistent low estimated glomerular filtration rate (eGFR<60 ml/min/1.73m^2^) (1, 2). CKD ranked in the top 30 of all diseases for several metrics globally and moved up to 18 in ranking for disability adjusted life years (DALY) from 1990 to 2019 (3). CKD is influenced by various risk factors including hypertension, diabetes, high sodium diet, and lead exposure (4), with diabetes being one of the primary causes of death in CKD (5). Moreover, diabetes is also the most common causes of the DALYs in CKD. The incidence of type 2 diabetes related chronic kidney disease (CKD-T2DM) in 2017 had increased by 74% compared with 1990 (6). Besides, there were 2.5 million cases of type 2 diabetes related chronic kidney disease (CKD-T2DM) in 2019, compared to 0.01 million cases of type 1 diabetes related chronic kidney disease globally (7), highlighting the significant contribution of type 2 diabetes to CKD burden.

CKD-T2DM is characterized by glomerular basement membrane thickening, mesangial expansion, nodular glomerular sclerosis, and renal tubular interstitial fibrosis (8), exhibiting considerable spatial and temporal variability worldwide (7). Age-standardized incidence rates (ASIR) of CKD-T2DM are closely associated with socio-demographic indexes (SDI) (6), with regions of higher SDI showing higher incidence rates but slower rates of increase since 1990. In 2019, East Asia and South Asia accounted for the most incident cases, while North Africa and Middle East exhibited the highest incidence rate, and Andean Latin America displayed the fastest increasing rate (7).

Numerous studies (9–11) have assessed the burden of DALYs and deaths of CKD attributable to CKD in China. By 2017, CKD had risen to become the 21^st^ leading cause of DALYs (9). During the past three decades, CKD-T2DM was responsible for the rapid increase of deaths in CKD. In 2019, CKD-T2DM was related to 32,000 deaths in women and similar results observed in men (10). However, a downward trend in mortality rate of CKD-T2DM was observed, attributed to population growth and aging (11).

Despite the growing number of studies reporting on the global burden of CKD (12–14), limited literature exists evaluating the sex-age-specific incidence rate of CKD-T2DM in different regions, SDI, and within China. Therefore, we aimed to evaluate the spatial-temporal changes in the incidence of CKD-T2DM from 1990 to 2019, categorized by age and sex in 21 regions with different SDI, and to predict the global incidence rate between 2020 and 2030 in this study.

## 2 Materials and methods

### 2.1 Data sources

The study data was retrieved from Global Burden of Disease Study 2019 (GBD2019) using the Global Health Data Exchange (GHDx) query tool (http://ghdx.healthdata.org/gbd-results-tool/). GBD2019 is an open access database that include data of the incidence, prevalence, death, DALYs, years of life lost (YLLs), years lived with disability (YLDs) for 369 diseases and injuries in 204 countries and territories (5). CKD is a non-communicable disease that can be influenced by various risk factors (4). The GBD2019 database mentioned five types of CKD, including CKD-T1DM, CKD-T2DM, CKD caused by hypertension, glomerulonephritis and other unspecified reasons. CKD-T2DM is one of the most serious complications of type 2 diabetes. The ICD-10 codes used to identify CKD-T2DM in this study were E11.22 (https://icdlist.com/icd-10/codes/type-2-diabetes-mellitus-e11). And all data utilized in this study was modeled estimates from the GBD 2019 database rather than raw data directly, to ensure comparability across regions and years. We estimated the age-standardized incidence rate (ASIR) of CKD-T2DM and its changing trends in 21 GBD regions, 5 SDI regions and also in China from 1990 to 2019. The population projections were extracted from World Population Prospects 2022(https://population.un.org/wpp/Download/Standard/CSV/). The age-standardized rate of population was obtained from the World Standard (WHO 2000-2025) (https://seer.cancer.gov/stdpopulations/world.who.html).

### 2.2 Covariates

We categorized the ASIR into 6 subgroups by sex (males and females) and age (15-49, 50-74, ≥75). SDI, ranging from 0-1, serves as an index measuring the social development. It includes indicators such as the fertility rate of women under 25, education level of people aged 15 and above, and the lag of per capita income distribution in a country. Regions were grouped by SDI based on the GBD 2019 criteria, categorizing them into five levels: High SDI (>0.81), High-middle SDI (0.70-0.81), Middle SDI (0.61-0.69), Low-middle SDI (0.46-0.60), and Low SDI (<0.46).

### 2.3 Statistical analysis

To eliminate the differences in age structure, we use ASIR to estimate the CKD-T2DM burden. Besides, we employed average annual percentage change (AAPCs) and its 95% confidence interval (95%CI) to evaluate the overall changing trends of CKD-T2DM in different regions for the past 3 decades. The annual percentage change (APCs) represented the rates change per year and fitted using a simple linear regression model. AAPCs were utilized when the rates change was not constant over a given time period (15). The AAPCs and APCs were calculated using the Joinpoint software, with detailed calculation available in other research (15, 16). We employed Joinpoint regression analysis to calculate AAPC and allowed a maximum of five joinpoints in the analysis. The model selection was based on the permutation test, which evaluates the statistical significance of each potential joinpoint and selects the optimal model. Region, age, and sex were stratified to identify variations in trends across different subgroups.

In addition, the Pearson correlation coefficient was calculated to estimate the correlation between SDI and ASIR in 3 age groups by sex and locally weighted regression was also performed to obtain the expected relationship between SDI and ASIR.

Age-period-cohort analysis was used to explain the decomposition effects of age, period and birth cohort on the incidence rate of CKD-T2DM, and it includes local drift, fitted longitudinal age-specific rates and period/cohort rate ratio compared with the reference period/cohort (17). Furthermore, we employed the Bayesian age-period-cohort (BAPC) model to project CKD-T2DM incidence using software R (version 4.2.1). The BAPC model was implemented using Integrated Nested Laplace Approximations (INLA), which allows for computationally efficient Bayesian inference without resorting to Markov Chain Monte Carlo (MCMC) methods. This makes the model suitable for routine applications involving large-scale datasets (18). Moreover, this model performed favorably against alternatives like the generalized Lee-Carter model, with lower absolute prediction errors and better continuous ranked probability scores. The BAPC model decomposes the incidence rate into three components: age effects, period effects, and cohort effects. We used weakly informative priors to ensure that the data drives the inference while maintaining stability in the estimation process. Convergence was assessed using trace plots and R-hat values, with all R-hat values below 1.1, indicating satisfactory convergence. Model validation was performed through out-of-sample predictions, with low root mean squared error (RMSE) and continuous ranked probability score (CRPS) values demonstrating good predictive accuracy. We incorporated population projections between 2020 and 2030, the age-standardized rate of the population, incidence data of CKD-T2DM from 1990 to 2019 and calculated the incidence cases and rates of CKD-T2DM by 5 years old age group globally and also in China. The significance level was 0.05 in this study.

## 3 Results

### 3.1 Global ASIR of CKD-T2DM and its changing trends

The new incidence cases and ASIR of CKD-T2DM by age and regions from 1990 to 2019 and the AAPCs in males and females were shown in **Tables 1**, **2** and **Supplementary Tables S1**-**S4**. In 2019, the global incidence of CKD-T2DM increased substantially across all age groups. Among the 21 GBD regions, East Asia reported the highest number of new cases of CKD-T2DM in both males and females under the age of 75, particularly within the 50–74 age group, which accounted for 160.84 thousand and 149.93 thousand cases. South Asia ranked second in terms of new cases in 2019. Among individuals aged above 75 years, Central Europe and East Asia recorded the largest number of CKD-T2DM incident cases.

**Table 1:** Global and regional incidence cases and ASIR of CKD-T2DM from 1990 to 2019 in males aged 15–49 years.

Location	Incidence cases (No.×1000)		ASIR		
	1990	2019	1990	2019	AAPC
Global	42.63 (34.07-52.61)	92.76 (74.01-114.78)	3.10 (2.48-3.83)	4.66 (3.72-5.77)	1.42 (1.35-1.49)
Andean Latin America	0.23 (0.17-0.30)	0.86 (0.64-1.11)	2.51 (1.88-3.26)	5.18 (3.85-6.72)	2.51 (2.48-2.55)
Australasia	0.07 (0.04-0.10)	0.15 (0.10-0.21)	1.25 (0.83-1.77)	2.19 (1.42-3.06)	1.96 (1.6-2.31)
Caribbean	0.40 (0.32-0.52)	1.00 (0.79-1.24)	4.53 (3.59-5.77)	8.41 (6.65-10.49)	2.16 (2.08-2.24)
Central Asia	0.51 (0.38-0.66)	1.38 (1.03-1.79)	3.06 (2.28-4.01)	5.65 (4.22-7.30)	2.15 (2.08-2.22)
Central Europe	1.11 (0.86-1.40)	1.47 (1.11-1.88)	3.59 (2.8-4.53)	5.46 (4.12-7.01)	1.45 (1.36-1.53)
Central Latin America	2.55 (2.02-3.15)	9.37 (7.64-11.35)	6.43 (5.09-7.93)	14.58 (11.9-17.67)	2.85 (2.80-2.91)
Central Sub-Saharan Africa	0.14 (0.10-0.19)	0.48 (0.34-0.67)	1.16 (0.82-1.61)	1.56 (1.1-2.17)	1.01 (0.93-1.08)
East Asia	8.30 (6.50-10.29)	14.18 (10.81-18.02)	2.33 (1.82-2.89)	3.70 (2.82-4.71)	1.62 (1.54-1.71)
Eastern Europe	2.02 (1.56-2.54)	3.65 (2.76-4.63)	3.68 (2.83-4.62)	7.49 (5.65-9.5)	2.51 (2.43-2.6)
Eastern Sub-Saharan Africa	0.44 (0.34-0.55)	1.27 (0.97-1.61)	1.09 (0.84-1.38)	1.30 (0.99-1.65)	0.61 (0.56-0.67)
High-income Asia Pacific	2.36 (1.84-2.95)	2.62 (2.01-3.32)	5.01 (3.9-6.26)	6.28 (4.81-7.94)	0.82 (0.71-0.94)
High-income North America	2.12 (1.54-2.78)	3.00 (2.23-3.92)	2.85 (2.07-3.73)	3.58 (2.66-4.68)	0.85 (0.47-1.23)
North Africa and Middle East	2.81 (2.21-3.5)	13.70 (10.64-17.04)	3.38 (2.65-4.2)	7.84 (6.09-9.74)	2.94 (2.89-2.99)
Oceania	0.06 (0.04-0.07)	0.15 (0.11-0.20)	3.46 (2.61-4.52)	4.41 (3.25-5.86)	0.83 (0.80-0.86)
South Asia	11.19 (8.98-13.74)	19.99 (15.9-24.81)	4.08 (3.27-5.01)	4.02 (3.20-5.00)	-0.03 (-0.1-0.05)
Southeast Asia	3.63 (2.88-4.54)	10.23 (8.11-12.71)	3.12 (2.47-3.9)	5.60 (4.44-6.96)	2.03 (1.96-2.1)
Southern Latin America	0.29 (0.21-0.38)	0.56 (0.4-0.75)	2.37 (1.71-3.11)	3.31 (2.39-4.43)	1.15 (1.09-1.20)
Southern Sub-Saharan Africa	0.43 (0.33-0.53)	1.02 (0.81-1.27)	3.38 (2.63-4.24)	4.84 (3.83-6.04)	1.20 (1.01-1.39)
Tropical Latin America	1.74 (1.38-2.12)	3.97 (3.17-4.91)	4.5 (3.57-5.49)	6.75 (5.38-8.33)	1.40 (1.35-1.45)
Western Europe	1.74 (1.24-2.33)	2.07 (1.46-2.74)	1.78 (1.26-2.39)	2.14 (1.51-2.84)	0.64 (0.53-0.75)
Western Sub-Saharan Africa	0.49 (0.38-0.62)	1.65 (1.26-2.10)	1.17 (0.9-1.48)	1.61 (1.22-2.05)	1.08 (1.01-1.15)
China	7.88 (6.16-9.82)	13.42 (10.21-17.13)	2.28 (1.78-2.84)	3.63 (2.76-4.63)	1.63 (1.54-1.72)

ASIR, age-standardized incidence rate; CKD-T2DM, type 2 diabetes related chronic kidney disease; AAPC, annual average percentage changes.

**Table 2:** Global and regional incidence cases and ASIR of CKD-T2DM from 1990 to 2019 in females aged 15–49 years.

Location	Incidence cases (No.×1000)		ASIR		
	1990	2019	1990	2019	AAPC
Global	40.77 (32.96-50.31)	83.09 (66.25-102.09)	3.05 (2.46-3.76)	4.27 (3.41-5.25)	1.18 (1.13-1.23)
Andean Latin America	0.23 (0.17-0.29)	0.81 (0.6-1.04)	2.38 (1.81-3.03)	4.9 (3.62-6.25)	2.49 (2.44-2.55)
Australasia	0.10 (0.07-0.14)	0.17 (0.12-0.24)	1.89 (1.34-2.53)	2.57 (1.76-3.53)	1.08 (0.91-1.25)
Caribbean	0.31 (0.24-0.40)	0.72 (0.56-0.92)	3.37 (2.61-4.29)	6.00 (4.64-7.65)	2.01 (1.98-2.04)
Central Asia	0.57 (0.43-0.74)	1.34 (0.99-1.74)	3.38 (2.55-4.38)	5.5 (4.06-7.14)	1.71 (1.61-1.8)
Central Europe	1.04 (0.8-1.34)	1.3 (0.97-1.66)	3.46 (2.64-4.42)	5.02 (3.77-6.45)	1.32 (1.21-1.43)
Central Latin America	1.9 (1.49-2.38)	6.06 (4.92-7.37)	4.54 (3.56-5.68)	8.98 (7.29-10.93)	2.37 (2.28-2.45)
Central Sub-Saharan Africa	0.17 (0.12-0.23)	0.53 (0.38-0.72)	1.35 (0.95-1.84)	1.71 (1.22-2.29)	0.83 (0.77-0.88)
East Asia	7.94 (6.25-9.88)	12.95 (10.01-16.3)	2.38 (1.87-2.96)	3.56 (2.75-4.49)	1.42 (1.33-1.5)
Eastern Europe	2.56 (2.02-3.13)	3.65 (2.76-4.59)	4.62 (3.65-5.66)	7.41 (5.6-9.3)	1.65 (1.52-1.79)
Eastern Sub-Saharan Africa	0.49 (0.38-0.61)	1.3 (1.01-1.65)	1.14 (0.89-1.43)	1.28 (0.99-1.62)	0.39 (0.31-0.47)
High-income Asia Pacific	1.54 (1.17-1.96)	1.54 (1.13-1.99)	3.36 (2.55-4.29)	3.9 (2.88-5.06)	0.55 (0.37-0.73)
High-income North America	2.96 (2.26-3.75)	3.83 (3.01-4.79)	4.00 (3.05-5.06)	4.61 (3.62-5.76)	0.51 (0.38-0.64)
North Africa and Middle East	3.11 (2.42-3.87)	12.4 (9.7-15.39)	3.94 (3.07-4.89)	7.81 (6.11-9.69)	2.40 (2.36-2.44)
Oceania	0.05 (0.04-0.06)	0.12 (0.09-0.16)	3.08 (2.34-3.97)	3.60 (2.72-4.7)	0.53 (0.49-0.57)
South Asia	9.13 (7.44-11.12)	17.08 (13.77-21.13)	3.58 (2.92-4.36)	3.58 (2.88-4.43)	0.00 (-0.15-0.16)
Southeast Asia	3.66 (2.91-4.53)	9.16 (7.17-11.38)	3.05 (2.42-3.78)	5.10 (4-6.34)	1.8 (1.73-1.87)
Southern Latin America	0.32 (0.23-0.42)	0.62 (0.47-0.81)	2.58 (1.84-3.4)	3.62 (2.75-4.75)	1.18 (1.14-1.22)
Southern Sub-Saharan Africa	0.32 (0.24-0.41)	0.74 (0.57-0.94)	2.37 (1.81-3.03)	3.49 (2.67-4.4)	1.34 (1.29-1.38)
Tropical Latin America	1.41 (1.12-1.73)	3.16 (2.54-3.9)	3.54 (2.81-4.34)	5.23 (4.21-6.47)	1.32 (1.17-1.48)
Western Europe	1.88 (1.36-2.47)	2.31 (1.64-3.05)	1.96 (1.42-2.58)	2.46 (1.75-3.24)	0.79 (0.72-0.86)
Western Sub-Saharan Africa	1.08 (0.87-1.30)	3.3 (2.63-4.01)	2.48 (2.00-3.00)	2.94 (2.34-3.57)	0.58 (0.37-0.79)
China	7.57 (5.93-9.39)	12.24 (9.44-15.49)	2.34 (1.84-2.91)	3.49 (2.69-4.41)	1.39 (1.31-1.48)

ASIR, age-standardized incidence rate; CKD-T2DM, type 2 diabetes related chronic kidney disease; AAPC, annual average percentage changes.

The global ASIR of CKD-T2DM was significantly high in males and the older adult in 2019, and increased in all age groups since 1990. The ASIR was the highest among males aged 75 and above, reaching 262.46 per 100,000 people in 2019, but with the lowest increase rate over the past 30 years (AAPC=0.64, 95%CI: [0.61-0.66]). Similar results were found in females aged 75 and above (AAPC=0.30, 95%: [0.27-0.33]). In 2019, the highest ASIR of CKD-T2DM was observed among males aged 75 and above in Australasia (438.59), followed by Southern Latin America (402.19), High-income Asia Pacific (397.49), and High-income North America (382.61). Among females in the same age group, Southern Latin America reported the highest ASIR at 313.25, followed by Western Europe (300.52), North Africa and Middle East (299.00), and High-income North America (287.20). Conversely, the lowest ASIR of CKD-T2DM was observed among males aged 15–49 in Eastern Sub-Saharan Africa (1.30), followed by Central Sub-Saharan Africa (1.56) and Western Sub-Saharan Africa (1.61). Similarly, among females aged 15-49, the lowest ASIR was reported in Eastern Sub-Saharan Africa (1.28), Central Sub-Saharan Africa (1.71) and Western Europe (2.46).

From 1990 to 2019, the AAPCs of ASIR remained stable for males in South Asia (15–49 age group), High-income North America, Southern Latin America, Tropical Latin America (50–74 age group), as well as for females in South Asia (15–49 age group), Australasia (50–74 age group). A declining trend was only found among females in Eastern Europe (75 age group). Except for the above regions mentioned, an increasing trend in ASIR of CKD-T2DM was observed in all other regions for the three age groups. The most significant increase in ASIR of CKD-T2DM was found among males aged 15–49 in North Africa and Middle East (AAPC=2.94, 95%CI: [2.89-2.99]), and Central Latin America (AAPC=2.85, 95%CI: [2.80-2.91]). Similarly, among females, the most significant increase was observed in the 50–74 age group in Andean Latin America (AAPC=2.49, 95%CI: [2.44-2.55]).

### 3.2 CKD-T2DM ASIR and its changing trends by SDI

**Figure 1:**
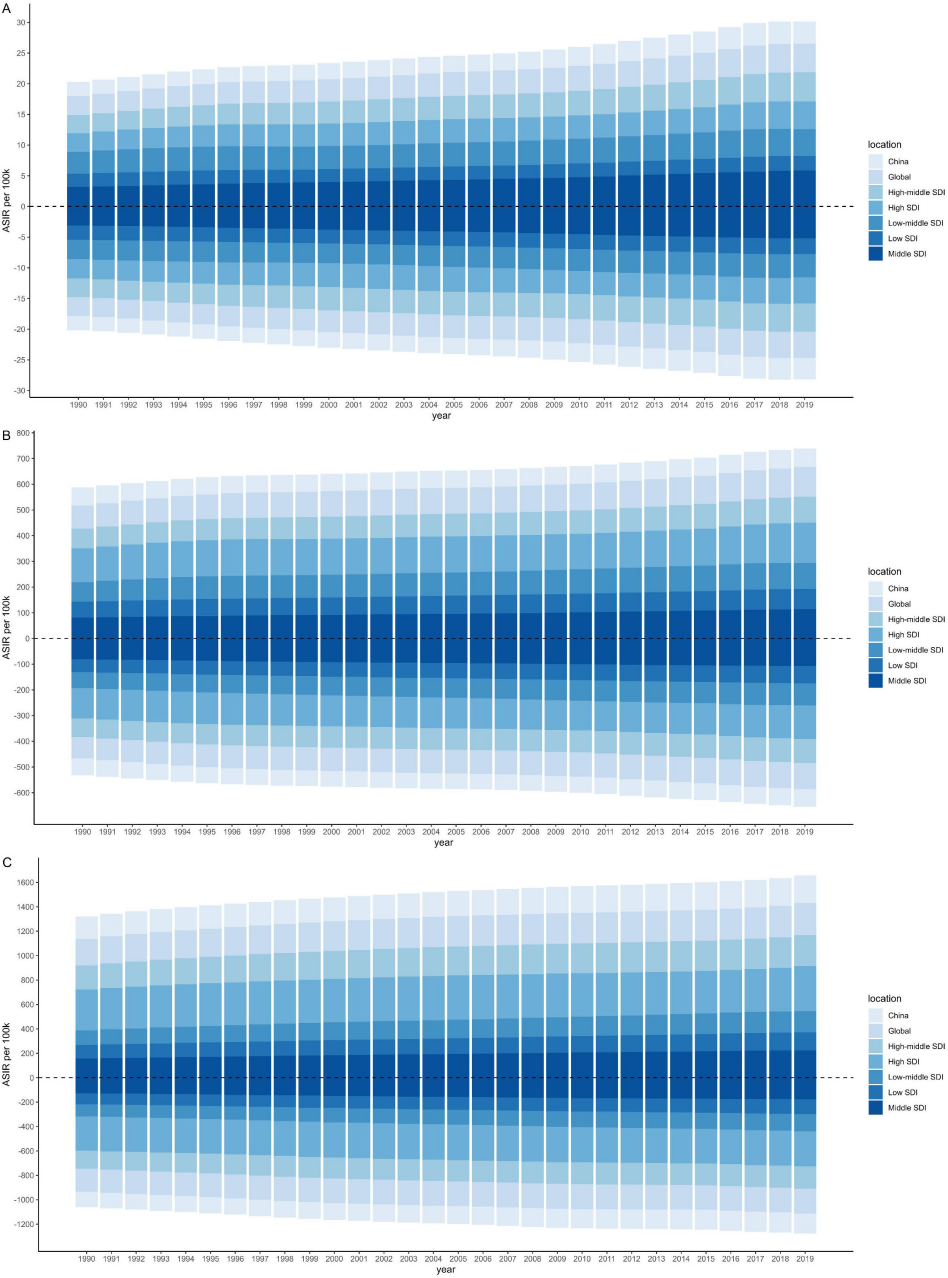
Age-standardized incidence rate per 100k by sociodemographic index (SDI), age and sex from 1990 to 2019. The upper column in each year is data for males, and the below for females. [**(A)** 15–49 years; **(B)** 50–74 years; **(C)** 75 plus].

In 2019, the highest ASIR was observed in males (368.97) (**Supplementary Table S5**) and females (287.52) (**Supplementary Table S6**) for the older 75 age group in High SDI region. The ASIR increased with SDI, except for the 15–49 age group where the highest ASIR was noted in Middle SDI region (**Figure 1**). Females was found to be higher ASIR than males only in the 15–49 age group in Low SDI region. From 1990 to 2019, the most significant increases were observed in both males (AAPC=2.11,95%CI: [2.06-2.16]) and females (AAPC= 1.80, 95%CI: [1.76-1.84]) in Middle SDI region for the 15–49 age group.

**Figure 2:**
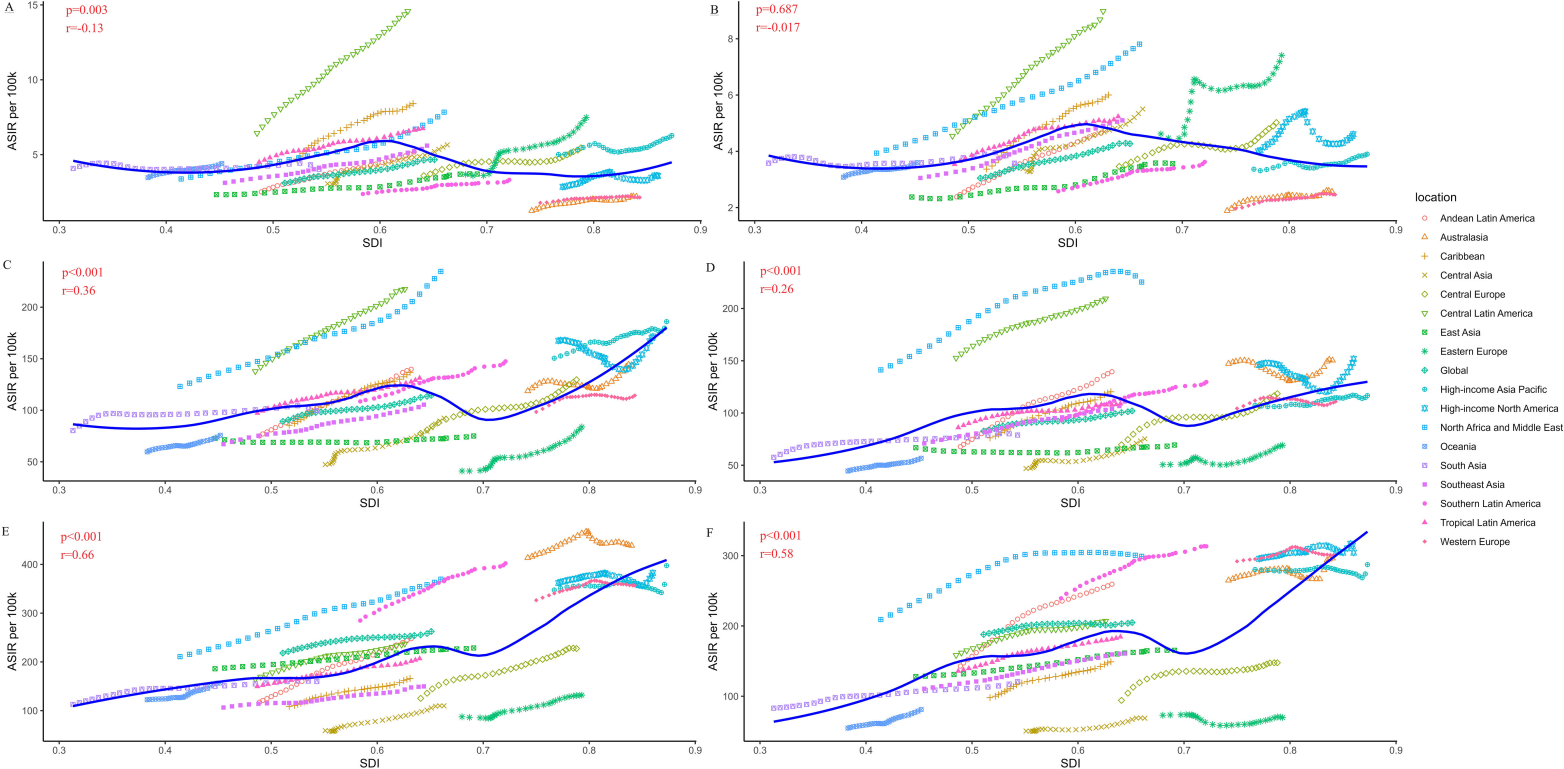
Age-standardized incidence rate (ASIR per 100k) of type 2 diabetes related chronic kidney disease (CKD-T2DM) in 21 regions by sex and age based on social-demographic index (SDI) from 1990 to 2019. Each combination of colors and shapes represents a region. The solid blue line represents the expected value within the range of entire SDI. The Pearson correlation coefficient and p-value are displayed. **(A)** ASIR per 100,000 population in males (15–49 years); **(B)** ASIR per 100,000 population in females (15–49 years); **(C)** ASIR per 100,000 population in males (50–74 years); **(D)** ASIR per 100,000 population in females (50–74 years); **(E)** ASIR per 100,000 population in males (≥75 years); **(F)** ASIR per 100,000 population in females (≥75 years).

**Figure 2** showed the variation of CKD-T2DM ASIR with the increase of SDI value by age and sex among 21 regions. The significant negative relationship between ASIR and SDI was only found in males for the 15–49 age group. Besides, ASIR showed moderately positive correlation relationship with SDI in both sex for the age group over 75 years. Overall, the ASIR increased with SDI value. According to the result of the locally weighted regression, the ASIR of CKD-T2DM in Central Latin America, North Africa and Middle East were much higher than the estimated value in almost all age groups. However, the ASIR of East Asia and Central Asia were found to be lower than the estimated value.

### 3.3 CKD-T2DM ASIR and its changing trends in China

China contributed substantially to the regional burden, accounting for over 90% of all new CKD-T2DM cases in East Asia across nearly all age groups. The ASIR and its changing trends in China were similar to globally, however, due to its large population base, the ASIR in China remained below the global average (**Figure 1**). ASIR of CKD-T2DM increased with age in both sexes, peaking in the older 75 age group, with rates reaching 227.09 in males and 163.86 in females in 2019 (**Tables 1**, **2** and **Supplementary Tables S1-S4**). During the past three decades, the most rapid increase in ASIR was observed in the 15–49 age group, with males showing an AAPC of 1.63 (95%CI: 1.54-1.72) and females an AAPC of 1.39 (95%CI: 1.31-1.48).

**Figure 3:**
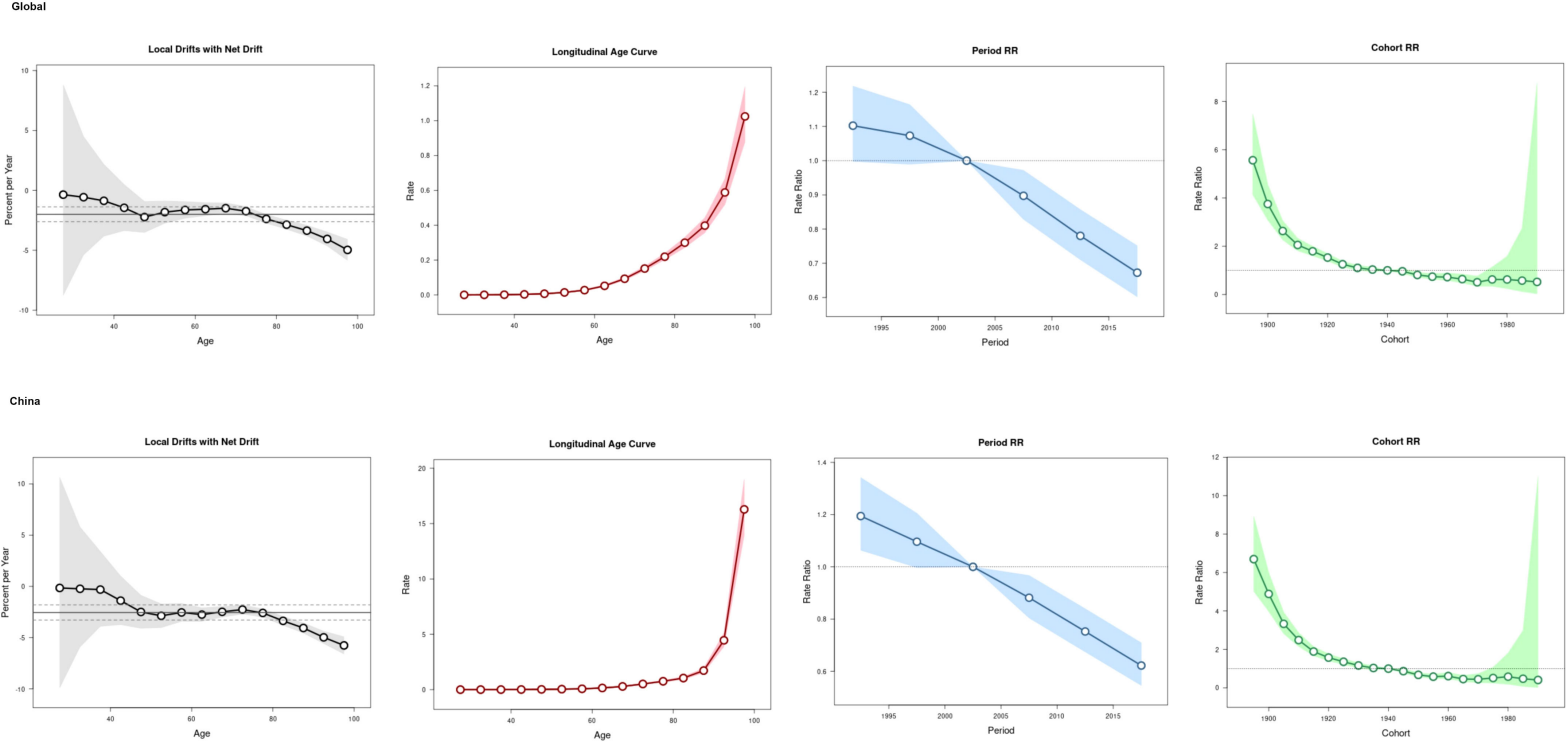
The age-period-cohort analysis of CKD-T2DM incidence globally and in China.

The age-period-cohort analysis (**Figure 3**) showed an increase trend of the ASIR with the increase of age, with a rapid increase found in the population aged above 60 years globally and over 80 years in China. Compared to period interval 2000-2004, the risk of CKD-T2DM incidence declined after 2005.The overall cohort effect was with the same patterns both globally and in China, the risk of incidence was higher in birth cohort before 1940 than those after 1940.

### 3.4 Prediction of ASIR of CKD-T2DM Using BAPC Model

**Figure 4:**
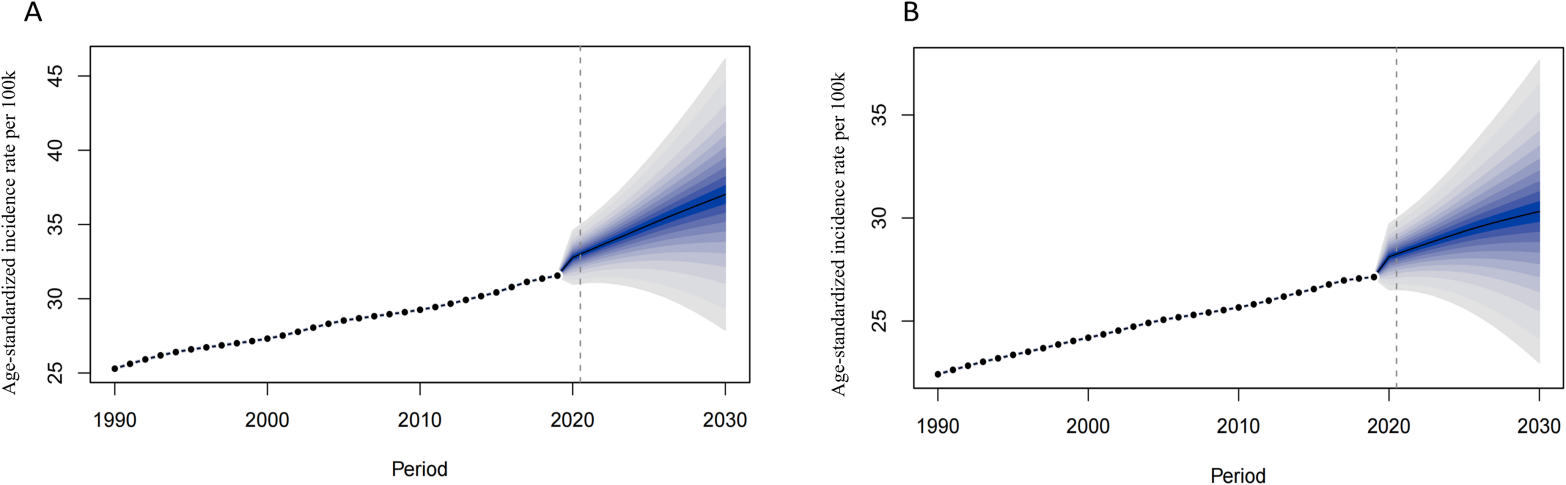
The prediction of CKD-T2DM incidence rate globally from 2020 to 2030. [**(A)** Males, **(B)** Females].

**Figure 5:**
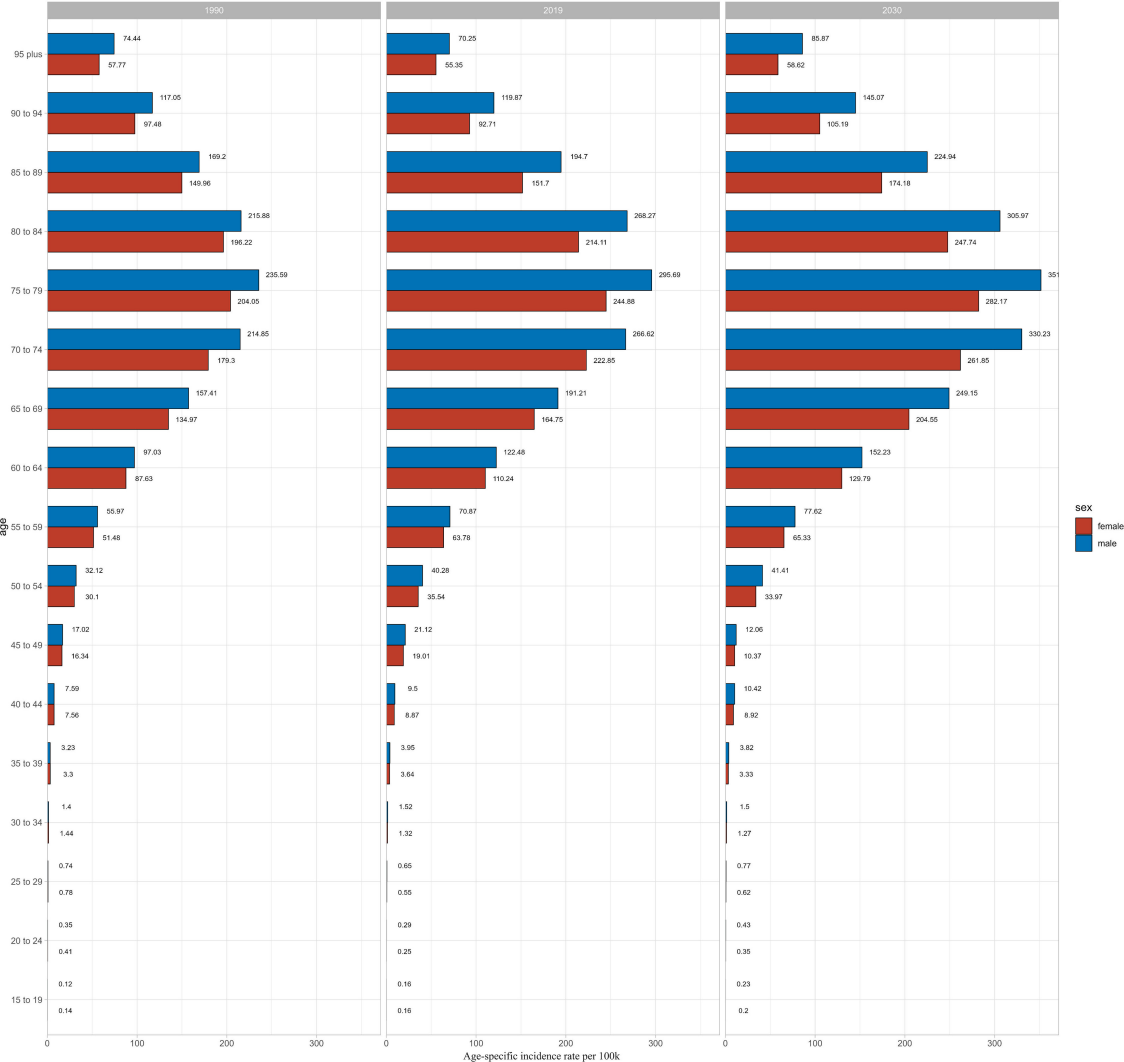
The prediction of global CKD-T2DM incidence rate by age and sex in 1990, 2019 and 2030.

We used BAPC model to predict the ASIR of CKD-T2DM from 2020 to 2030 by age and sex. Our findings revealed an overall increase in both the number of incidence cases and incidence rate globally, with the exception of age groups under 50 years in both sexes (**Supplementary Tables S7**-**S10**). The prediction of global ASIR was showed in **Figure 4**. The ASIR in males is predicted to increase from 25.29 to 37.02 per 100,000 between 1990 and 2030, while in females, it is expected to increase from 22.41 to 30.32 per 100,000. The highest number of incidence cases were found in 70–74 age group in both sexes. However, the incidence rate of CKD-T2DM was highest in the older 75–79 age group for both sex with 351.64 per 100k in males and 282.17 per 100k in females (**Figure 5**), indicating about 19 percent increase in males and 15 percent increase in females since 1990. Moreover, higher incidence cases and rates were found in age groups over 50 years.

Besides, we also conducted the prediction in China. As showed in **Supplementary Figures S1** and **S2**, the prediction of ASIR patterns were similar to global one, continued to increase since 2019. The highest incidence cases were found in 65–69 age group in both sexes, with highest ASIR in 75–79 age group (**Supplementary Tables S11**-**S14**). Higher incidence cases and rates were also showed in people aged over 50 years.

## 4 Discussion

Over the past three decades, CKD-T2DM has showed a notably high global incidence rate. Utilizing the latest CKD burden data in GBD2019, we estimated the ASIR of CKD-T2DM by sex, age and regions with different SDI and also in China. Consistent with previous studies (6, 7), we observed a significant increase in ASIR of CKD-T2DM with age and sex from 1990 to 2019. Furthermore, our projections indicated a continuing increase trend in ASIR of CKD-T2DM between 2020 and 2030.

In this study, we found that almost all regions showed the highest ASIR in the older adult age group (≥75), with projections indicating even higher rates in the older adult between 2020 and 2030. Besides, the age-period-cohort analysis revealed that the increase of CKD-T2DM incidence rate was significantly related to the rising age. Many studies have reported that advanced age, high blood pressure, Hyperlipidemia, smoking, high sodium diet, high body mass index, lead exposure, non-optimal temperature and some biomarkers such as kidney injury molecule 1 (KIM-1) and tumor necrosis factor receptors 2(TNFR-2) were the risk factors of CKD (4, 19, 20). The higher ASIR in the older adult may result from a cumulative effect of environmental and behavioral factors, compounded by population aging, which is a significant driver of CKD burden (1).

Furthermore, we observed a pronounced ASIR of CKD-T2DM in males compared to females in most regions. This phenomenon can be explained by the variation of hormones, which play an important role in the development of diabetes and CKD. Estrogen, for instance, confers protection against renal dysfunction in premenopausal and nondiabetic women, but loses efficacy post-menopause and in diabetic conditions (21), with this protective effect attenuating progressively with age (22). Conversely, biological factors including androgen-mediated susceptibility and visceral adiposity in males heighten their vulnerability (23, 24). Gender disparities are further compounded by paradoxical observations: although women exhibit higher obesity prevalence, they demonstrate lower T2DM risk compared to men (25). Behavioral differences significantly contribute, evidenced by higher male prevalence of smoking, unhealthy dietary patterns, and delayed healthcare-seeking (16, 23, 24, 26). Environmental exposures—particularly occupational PM2.5 affecting males disproportionately—constitute additional risk factors for diabetes and CKD (16). Systemic barriers, including reduced male participation in preventive screenings (e.g., urine ACR) and routine primary care, further widen this gap (27). Notably, rising CKD-T2DM incidence in the 15–49 age group is primarily driven by earlier onset of obesity and T2DM attributable to poor nutrition, sedentary lifestyles, and excessive sugar-sweetened beverage consumption (28, 29). Collectively, these biological, behavioral, environmental, and healthcare-access factors elucidate the observed gender disparities in CKD-T2DM incidence.

In 2019, Central Sub-Saharan Africa, Western Sub-Saharan Africa, and eastern Sub-Saharan Africa were all showed low incidence rate for the youngest age group. Several reasons may account for these results. Studies have reported the low incidence and prevalence rate of diabetes in Sub-Saharan Africa (30). However, the DALYs and deaths caused by diabetes were still high. The development of Sub-Saharan Africa was backward with low SDI. Although there was slightly increase in SDI in the past 30 years, it lags behind other regions (7). Additionally, South Africa, for instance, has a significantly low number of nephrologists, with only 2.3 per million individuals, far below the global average (31). Moreover, insufficient medical resources, backward detection, prevention, and treatment measures, such as renal replacement therapy and kidney transplantation, contribute to the burden of CKD (32). In contrast, Australasia, High-income north America and North Africa and Middle East showed high incidence rate. Previous studies also showed that the CKD incidence rate was high in these regions (7). In North Africa and the Middle East, previous studies on CKD have primarily focused on metrics such as age-standardized DALYs (ASDR) and age-standardized mortality rates (ASMR). Although certain behavioral and environmental factors such as high dietary sodium intake and exposure to non-optimal temperatures were associated with relatively modest attributable burdens, they remain important modifiable risk factors. Besides, factors such as malnutrition, smoking, air pollution, sandstorms, water scarcity, and rising temperatures may contribute significantly to the burden of CKD in North Africa and Middle East (33). In Central Latin America, our study identified a notably high and rapidly increasing ASIR of CKD-T2DM. This trend is consistent with findings from GBD2021 study, which emphasized the role of unhealthy dietary patterns such as low intake of fruits, high consumption of red meat, and frequent consumption of processed meats as major contributors to the CKD-T2DM. Furthermore, the study of GBD2021 reported substantial regional disparities in CKD-T2DM burden, with Central Latin America exhibiting particularly elevated ASMR and ASDR (34).

High SDI regions consistently showed high incidence rates in middle and older adult age groups, but with lower ASIR in the 15–49 years age group, partly due to improved medical facilities, greater investment in healthcare, advanced CKD detection technologies, and higher health literacy among residents (35), which together facilitate early detection and diagnosis of CKD-T2DM in younger populations. This enables timely intervention to slow or prevent disease progression in high-risk individuals. In contrast, lower diagnostic rates and limited diagnostic capacity in low-SDI regions may contribute to substantial underdiagnosis, particularly among younger individuals, potentially masking the true burden of CKD-T2DM in these areas. The availability of more healthcare professionals in advanced development regions, such as high-income countries, is noteworthy, with a nephrologist density of 28.52 per million compared to only 0.31 per million in low-income countries, which is 2.2 times greater than the global average (36). However, these disparities make it challenging for individuals in lower development area to seek effective diagnosis and medical assistance. Besides, compared with low development area, advanced development regions or high-income countries are more likely to be obese because of the dietary difference. Meanwhile, obesity, on the other words, high body mass index is the main cause of DALYs and deaths in diabetes and CKD, especially in low income and low-middle income countries (4). In low SDI regions, CKD is often difficult to diagnose early, and individuals face challenges in accessing kidney replacement therapy or dialysis therapy. Although there were increasing use of dialysis therapy in many countries, the DALYs and deaths in low SDI area still increased (37).

The incidence rate and its changing trend of CKD-T2DM in China was similar to the global patterns. The ASIR was highest in individuals aged 75 and above, and substantially higher in males than females. This trend can be attributed to population growth and aging. Research indicates that for every year of age increase, the incidence of CKD in China increases by 1.048 times (38). The burden of CKD-T2DM reached a peak in 90 years old (10). We found a constant increase trend of the ASIR of CKD-T2DM in China. Moreover, an increase trend of the death in Chinese CKD-T2DM has also been stated ever (11). A lower CKD-T2DM ASIR was also found in China compared to global one. Chinese government has launched several major chronic disease prevention initiatives, such as the Healthy China Action (2019–2030), which emphasizes early screening, lifestyle interventions, and integrated management of diabetes and its complications (39, 40), including CKD. China has implemented extensive chronic disease prevention and control programs, including community-based diabetes management and screening initiatives, facilitating timely interventions that may reduce CKD progression (41). The development of other CKD-T2DM scoring models has further improved screening accuracy, enabling more precise early detection (42). Second, Public health investment and education campaigns in China increased awareness of diabetes and its complications, encouraging lifestyle changes and more frequent health monitoring (43). Moreover, China’s population age structure includes a relatively lower proportion of older adult individuals compared to some high-income countries. Since age is a strong risk factor for CKD, this demographic difference may partially explain the lower national ASIR (10). In addition, there is a growing emphasis on managing lifestyle-related risk factors, such as high body mass index and unhealthy diet patterns. As China’s health profile transitions with economic development, national efforts have increasingly focused on addressing these modifiable factors, potentially mitigating CKD onset in diabetic populations (44, 45). Although China has a high number of new CKD-T2DM cases, the ASIR remains relatively low, largely due to its large population base. China has lower CKD-T2DM ASIR than global average but remains rapid growing trends, therefore, it’s important for us to put more attention on the burden of CKD.

Recent studies based on the GBD2021 data have highlighted a continuous global increase in the burden of CKD over the past three decades. T2DM remains the leading cause of CKD worldwide, with the ASIR of CKD attributable to T2DM rising by approximately 21.0% from 1990 to 2021. Similarly, age-standardized mortality and DALYs due to CKD-T2DM have also shown substantial increases (46). Notably, Central Asia reported the highest prevalence of CKD in 2021, while China recorded the highest number of new CKD cases (47), underscoring the significant and growing public health burden in this region. These trends emphasize the urgent need for improved prevention, early detection, and management strategies targeting diabetes-related CKD, particularly in countries with rapidly increasing incidence.

Our study has several limitations. First, as a secondary analysis of modeled GBD data, our findings may be affected by variations in source data quality, especially in low-SDI regions with inadequate death registry coverage etc. Second, our projections through 2030 are based on the Bayesian age-period-cohort model, which assumes the continuation of past trends and cannot account for unforeseen disruptions (e.g., pandemics). Additionally, evolving clinical definitions of CKD-T2DM during 1990–2019 may introduce heterogeneity in case identification. In future research, we recommend incorporating relevant clinical data for validation to enhance the credibility of our findings.

## 5 Conclusions

In summary, this study provides updated estimates of the incidence of CKD-T2DM by age, sex, region, and SDI, based on GBD 2019 data. We observed a rising incidence across both sexes and nearly all age groups, with projections suggesting continued global and national increases over the next decade. Given the growing burden, especially in high-SDI regions, it is essential to implement SDI-specific strategies to improve early detection and clinical management. High-SDI countries should enhance routine screening and disease control, while low- and middle-SDI regions should focus on expanding access to care and timely diagnosis. These findings highlight the urgent need for context-specific prevention and intervention strategies to mitigate the future impact of CKD-T2DM.

## Data Availability

The datasets presented in this study can be found in online repositories. The names of the repository/repositories and accession number(s) can be found below: http://ghdx.healthdata.org/gbd-results-tool/.
