# Supplementary material for: The global incidence rate of type 2 diabetes related chronic kidney disease and predictions by Bayesian age-period-cohort analysis: findings from the Global Burden of Disease Study 2019
Source: Front Endocrinol (Lausanne). 2025 Aug 11;16:1429048. doi: 10.3389/fendo.2025.1429048 (PMC12375498; doi:10.3389/fendo.2025.1429048)
Supplement: Supplementary file 1 [file DataSheet1.pdf]

Supplementary Material

Table S1. Global and regional incidence cases and ASIR of CKD-T2DM from 1990 to 2019 in males aged 50-74 years.

Location	Incidence cases (No. × 1000)		ASIR		
	1990	2019	1990	2019	AAPC
Global	333.64(285.59-382.37)	874.78(753.76-991.42)	89.20 (76.35-102.22)	114.47 (98.63-129.73)	0.86 (0.84-0.89)
Andean Latin America	1.45(1.22-1.7)	7.1(6.06-8.24)	75.11 (63.18-88.23)	139.77 (119.32-162.26)	2.18 (2.11-2.24)
Australasia	2.33(2.01-2.67)	5.58(4.43-6.65)	118.67 (102.09-135.95)	143.71 (114.29-171.28)	0.67 (0.55-0.78)
Caribbean	1.98(1.7-2.3)	6.38(5.45-7.29)	85.49 (73.1-99.25)	136.71 (116.77-156.22)	1.64 (1.59-1.68)
Central Asia	2(1.6-2.45)	6.81(5.58-8.22)	47.31 (37.85-57.96)	92.38 (75.75-111.52)	2.36 (2.21-2.51)
Central Europe	9.89(8.37-11.6)	21.47(18.11-24.75)	77.48 (65.54-90.88)	129.65 (109.33-149.43)	1.79 (1.72-1.86)
Central Latin America	10.79(9.11-12.73)	45.82(39.55-52.38)	137.79 (116.3-162.52)	217.51 (187.74-248.69)	1.58 (1.52-1.65)
Central Sub-Saharan Africa	1.1(0.94-1.27)	3.05(2.61-3.53)	48 (41.26-55.83)	57.02 (48.81-65.83)	0.60 (0.54-0.65)
East Asia	66.71(56.8-76.89)	160.84(136.56-185)	72.68 (61.89-83.77)	75.03 (63.7-86.29)	0.11 (0.04-0.17)
Eastern Europe	9.18(7.37-11.13)	21.91(18.06-26.33)	41.10 (33.00-49.86)	83.80 (69.05-100.7)	2.52 (2.35-2.69)
Eastern Sub-Saharan Africa	3.77(3.24-4.29)	9.34(8.01-10.68)	49.28 (42.34-56.19)	58.41 (50.10-66.76)	0.58 (0.52-0.64)
High-income Asia Pacific	27.18(23.67-30.61)	55.43(47.27-63.74)	150.48 (131.01-169.48)	185.95 (158.55-213.82)	0.73 (0.58-0.88)
High-income North America	43.92(36.2-52.67)	88.04(73.66-102.93)	167.48 (138.06-200.87)	171.5 (143.49-200.5)	0.08 (-0.07-0.23)
North Africa and Middle East	22.07(19.12-25.43)	106.38(91.56-121.68)	123.13 (106.71-141.89)	234.68 (201.98-268.42)	2.26 (2.21-2.3)
Oceania	0.19(0.16-0.23)	0.6(0.51-0.71)	59.63 (49.85-69.94)	75.72 (63.51-88.73)	0.82 (0.79-0.85)
South Asia	50.36(42.58-58.63)	137.73(117.5-158.12)	80.34 (67.94-93.54)	99.63 (84.99-114.38)	0.76 (0.69-0.83)
Southeast Asia	16.77(14.16-19.42)	64.24(55.02-73.16)	67.15 (56.72-77.78)	105.39 (90.27-120.02)	1.54 (1.47-1.61)
Southern Latin America	4.35(3.64-5.06)	10.09(8.5-11.77)	108.58 (90.85-126.5)	147.39 (124.07-171.9)	1.05 (0.97-1.13)
Southern Sub-Saharan Africa	2.26(1.9-2.64)	6.5(5.59-7.41)	94.16 (79.41-110.33)	132.84 (114.27-151.41)	1.18 (1.07-1.29)
Tropical Latin America	8.37(7.12-9.65)	28.64(24.87-32.48)	97.24 (82.77-112.21)	131.25 (113.98-148.86)	1.03 (0.96-1.1)
Western Europe	43.73(36.72-50.65)	74.71(63.93-84.8)	98.43 (82.65-114.02)	114.34 (97.84-129.77)	0.52 (0.46-0.58)
Western Sub-Saharan Africa	5.27(4.58-6.05)	14.11(12.18-16.09)	56.2 (48.83-64.5)	76.91 (66.42-87.69)	1.10 (1.03-1.17)
China	62.86(53.22-72.64)	152.04(128.33-175.48)	71.00(60.11-82.05)	73.16 (61.75-84.44)	0.10 (0.01-0.19)

Abbreviations: ASIR: age-standardized incidence rate; CKD-T2DM: type 2 diabetes related chronic kidney disease; AAPC: annual average percentage changes.

Table S2. Global and regional incidence cases and ASIR of CKD-T2DM from 1990 to 2019 in females aged 50-74 years.

Location	Incidence cases (No. × 1000)		ASIR		
	1990	2019	1990	2019	AAPC
Global	324.69(276.05-374.31)	817.72(707.58-924.64)	89.20 (76.35-102.22)	114.47 (98.63-129.73)	0.86 (0.84-0.89)
Andean Latin America	1.34(1.12-1.57)	7.51(6.41-8.68)	75.11 (63.18-88.23)	139.77 (119.32-162.26)	2.18 (2.11-2.24)
Australasia	3.02(2.68-3.38)	6.18(5.21-7.14)	118.67 (102.09-135.95)	143.71 (114.29-171.28)	0.67 (0.55-0.78)
Caribbean	1.84(1.57-2.16)	6.04(5.21-6.93)	85.49 (73.1-99.25)	136.71 (116.77-156.22)	1.64 (1.59-1.68)
Central Asia	2.48(2.02-2.99)	6.59(5.4-7.85)	47.31 (37.85-57.96)	92.38 (75.75-111.52)	2.36 (2.21-2.51)
Central Europe	10.53(8.84-12.49)	22.09(18.61-25.66)	77.48 (65.54-90.88)	129.65 (109.33-149.43)	1.79 (1.72-1.86)
Central Latin America	12.62(10.8-14.83)	50.15(44.59-55.75)	137.79 (116.3-162.52)	217.51 (187.74-248.69)	1.58 (1.52-1.65)
Central Sub-Saharan Africa	0.93(0.78-1.1)	2.91(2.47-3.46)	48 (41.26-55.83)	57.02 (48.81-65.83)	0.60 (0.54-0.65)
East Asia	58.71(49.64-68.06)	149.93(129.17-170.27)	72.68 (61.89-83.77)	75.03 (63.7-86.29)	0.11 (0.04-0.17)
Eastern Europe	16.47(13.65-19.48)	24.63(20.62-29.01)	41.10 (33.00-49.86)	83.80 (69.05-100.7)	2.52 (2.35-2.69)
Eastern Sub-Saharan Africa	2.7(2.29-3.15)	7.8(6.59-9.12)	49.28 (42.34-56.19)	58.41 (50.10-66.76)	0.58 (0.52-0.64)
High-income Asia Pacific	22.06(18.61-25.28)	35.75(29.57-41.57)	150.48 (131.01-169.48)	185.95 (158.55-213.82)	0.73 (0.58-0.88)
High-income North America	43.97(36.42-52.16)	83.93(71.23-96.11)	167.48 (138.06-200.87)	171.5 (143.49-200.5)	0.08 (-0.07-0.23)
North Africa and Middle East	24.07(20.47-28.38)	96.34(83.22-110.66)	123.13 (106.71-141.89)	234.68 (201.98-268.42)	2.26 (2.21-2.3)
Oceania	0.13(0.11-0.16)	0.41(0.34-0.48)	59.63 (49.85-69.94)	75.72 (63.51-88.73)	0.82 (0.79-0.85)
South Asia	32.12(26.89-37.53)	109(92.83-126.37)	80.34 (67.94-93.54)	99.63 (84.99-114.38)	0.76 (0.69-0.83)
Southeast Asia	19.34(16.39-22.42)	73.51(63.75-84.05)	67.15 (56.72-77.78)	105.39 (90.27-120.02)	1.54 (1.47-1.61)
Southern Latin America	4.26(3.52-5.08)	10.01(8.39-11.69)	108.58 (90.85-126.5)	147.39 (124.07-171.9)	1.05 (0.97-1.13)
Southern Sub-Saharan Africa	1.83(1.52-2.2)	5.84(4.91-6.88)	94.16 (79.41-110.33)	132.84 (114.27-151.41)	1.18 (1.07-1.29)
Tropical Latin America	8.08(6.88-9.29)	27.07(23.3-30.77)	97.24 (82.77-112.21)	131.25 (113.98-148.86)	1.03 (0.96-1.1)
Western Europe	52.85(43.88-61.48)	76.39(64.48-88.09)	98.43 (82.65-114.02)	114.34 (97.84-129.77)	0.52 (0.46-0.58)
Western Sub-Saharan Africa	5.31(4.54-6.19)	15.63(13.29-18.01)	56.2 (48.83-64.5)	76.91 (66.42-87.69)	1.10 (1.03-1.17)
China	55.81(47.13-64.96)	141.36(121.06-161.11)	71.00(60.11-82.05)	73.16 (61.75-84.44)	0.10 (0.01-0.19)

Abbreviations: ASIR: age-standardized incidence rate; CKD-T2DM: type 2 diabetes related chronic kidney disease; AAPC: annual average percentage changes.

Table S3. Global and regional incidence cases and ASIR of CKD-T2DM from 1990 to 2019 in males aged above 75 years.

Location	Incidence cases (No.×1000)		ASIR		
	1990	2019	1990	2019	AAPC
Global	97.55(77.87-121.02)	302.64(247.17-368.35)	218.18 (174.17-270.67)	262.46 (214.35-319.44)	0.64 (0.61-0.66)
Andean Latin America	0.33(0.26-0.42)	2.2(1.75-2.8)	117.40 (91.81-146.72)	247.59 (196.9-314.91)	2.61 (2.57-2.66)
Australasia	1.36(1.11-1.65)	3.99(3.23-4.78)	413.53 (338.61-499.86)	438.59 (355.1-526.19)	0.20 (0.18-0.22)
Caribbean	0.44(0.34-0.57)	1.38(1.08-1.77)	108.64 (82.91-139.31)	166.26 (130.44-212.88)	1.49 (1.43-1.54)
Central Asia	0.27(0.2-0.35)	0.65(0.48-0.86)	59.05 (44.62-77.15)	109.88 (81.94-146.57)	2.18(2.02-2.33)
Central Europe	2.31(1.78-2.97)	7.06(5.58-8.91)	125.18 (96.25-160.84)	227.47 (179.86-287.1)	2.08 (2.01-2.14)
Central Latin America	1.82(1.4-2.33)	8.28(6.48-10.42)	164.69 (126.39-210.95)	237.3 (185.83-298.56)	1.26 (1.22-1.31)
Central Sub-Saharan Africa	0.16(0.12-0.2)	0.41(0.32-0.52)	89.07 (67.89-113.62)	107.51 (82.97-135.53)	0.65 (0.61-0.70)
East Asia	14.94(11.69-18.94)	61.32(48.92-76.14)	185.97 (145.54-235.8)	228.52 (182.31-283.79)	0.71 (0.70-0.72)
Eastern Europe	2.05(1.55-2.67)	4.79(3.7-6.16)	87.50 (66.33-114.15)	131.83 (101.88-169.3)	1.44 (1.35-1.52)
Eastern Sub-Saharan Africa	0.73(0.58-0.91)	1.83(1.43-2.3)	99.18 (78.97-124.78)	114.36 (89.52-143.55)	0.49 (0.47-0.50)
High-income Asia Pacific	8.64(7.09-10.56)	35.09(29.03-42.55)	347.39 (285.09-424.91)	397.49 (328.82-481.96)	0.40 (0.30-0.50)
High-income North America	18.67(14.7-23.08)	39.65(32.42-48.55)	361.96 (284.99-447.37)	382.61 (312.88-468.52)	0.19 (0.13-0.25)
North Africa and Middle East	3.92(3.06-4.93)	20.26(15.95-25.42)	210.78 (164.44-265.08)	369.65 (291.07-463.7)	1.95 (1.89-2)
Oceania	0.03(0.02-0.04)	0.1(0.08-0.12)	122.38 (95.79-156.2)	156.68 (122.2-202.68)	0.86 (0.69-1.03)
South Asia	6.28(4.79-8.17)	28.13(21.93-35.85)	112.8 (86.11-146.84)	159.82 (124.6-203.66)	1.21 (1.17-1.24)
Southeast Asia	2.66(2.05-3.43)	9.42(7.1-12.18)	106.45 (81.77-137.13)	149.78 (112.89-193.63)	1.18 (1.11-1.25)
Southern Latin America	1.71(1.37-2.17)	5.02(4.1-6.11)	284.89 (228.14-361.86)	402.19 (328.44-489.44)	1.18 (1.15-1.22)
Southern Sub-Saharan Africa	0.5(0.39-0.64)	1.22(0.96-1.54)	184.89 (143.97-233.86)	237.27 (186.99-299.12)	0.87 (0.82-0.92)
Tropical Latin America	1.55(1.19-2)	6.72(5.4-8.44)	149.62 (114.68-192.04)	206.82 (166.2-259.87)	1.11 (1.09-1.14)
Western Europe	28.02(22.84-34.41)	61.78(52.24-73.44)	326.53 (266.19-401.02)	356.82 (301.72-424.2)	0.30 (0.26-0.34)
Western Sub-Saharan Africa	1.16(0.94-1.43)	3.34(2.68-4.14)	126.86 (102.63-156.31)	159.58 (128.14-198.09)	0.79 (0.77-0.81)
China	14.18(11.03-18.04)	58.84(46.88-73.25)	182.66 (142.08-232.38)	227.09 (180.91-282.68)	0.75 (0.74-0.76)

Abbreviations: ASIR: age-standardized incidence rate; CKD-T2DM: type 2 diabetes related chronic kidney disease; AAPC: annual average percentage changes.

Table S4. Global and regional incidence cases and ASIR of CKD-T2DM from 1990 to 2019 in females aged above 75 years.

Location	Incidence cases (No. × 1000)		ASIR		
	1990	2019	1990	2019	AAPC
Global	135.89(109.74-166.99)	330.25(273.69-396.75)	187.89(151.73-230.9)	204.77(169.7-246)	0.30(0.27-0.33)
Andean Latin America	0.46(0.37-0.58)	2.65(2.13-3.32)	142.93(113.52-178.81)	259.34(208.5-324.94)	2.09(2.03-2.15)
Australasia	1.46(1.21-1.79)	3.42(2.81-4.24)	264.81(219.15-324.87)	296.25(243.12-366.87)	0.42(0.31-0.54)
Caribbean	0.46(0.36-0.61)	1.57(1.25-1.96)	98.03(75.74-128.18)	148.97(118.53-185.45)	1.47(1.42-1.52)
Central Asia	0.5(0.37-0.66)	0.75(0.56-1.02)	50.56(38.22-67.35)	68.78(50.63-93.45)	1.08(1.00-1.17)
Central Europe	3.08(2.35-4.05)	8.22(6.43-10.32)	94.14(71.74-123.6)	147.65(115.54-185.51)	1.57(1.38-1.76)
Central Latin America	2.02(1.55-2.59)	9.14(7.34-11.22)	158.39(121.81-203.62)	206.93(166.02-253.83)	0.93(0.88-0.99)
Central Sub-Saharan Africa	0.15(0.11-0.19)	0.74(0.57-0.93)	79.55(60.76-103.21)	110.61(85.43-139.64)	1.14(1.12-1.16)
East Asia	15.47(12.09-19.83)	59.21(48.25-72.4)	128.07(100.05-164.13)	165.35(134.75-202.19)	0.89(0.84-0.94)
Eastern Europe	5.45(4.17-7.07)	6.57(5.03-8.52)	72.97(55.87-94.69)	69.68(53.37-90.29)	-0.16(-0.24--0.09)
Eastern Sub-Saharan Africa	0.66(0.52-0.83)	2.18(1.73-2.72)	79.55(62.87-100.48)	104.98(83.39-130.85)	0.97(0.93-1.01)
High-income Asia Pacific	11.99(9.77-14.64)	38.79(32.61-45.82)	280.27(228.39-342.13)	287.20(241.4-339.19)	0.07(0.03-0.12)
High-income North America	27.65(22.27-33.36)	44.39(36.87-53.23)	295.52(238.08-356.56)	304.32(252.81-364.96)	0.11(0.01-0.21)
North Africa and Middle East	4.34(3.34-5.54)	16.85(13.02-21.44)	209.11(160.89-267.07)	299.00(230.93-380.45)	1.24(1.22-1.27)
Oceania	0.01(0.01-0.02)	0.06(0.04-0.07)	54.72(41.16-72.59)	81.1(62.64-106.03)	1.36(1.33-1.4)
South Asia	4.43(3.37-5.81)	24.56(18.96-31.24)	83.07(63.21-108.99)	120.84(93.27-153.69)	1.31(1.24-1.38)
Southeast Asia	3.78(2.92-4.86)	15.31(11.78-19.69)	110.64(85.38-142.15)	160.97(123.8-206.96)	1.30(1.27-1.34)
Southern Latin America	2.25(1.82-2.83)	6.25(5.21-7.53)	239.32(193.63-300.92)	313.25(261.05-377.58)	0.93(0.89-0.97)
Southern Sub-Saharan Africa	0.55(0.43-0.7)	1.59(1.25-2.02)	119.49(93.14-150.6)	161.08(126.73-204.02)	1.03(0.98-1.07)
Tropical Latin America	1.86(1.44-2.38)	8.75(7.16-10.85)	135.77(105.05-173.82)	184.5(150.87-228.71)	1.06(1.01-1.10)
Western Europe	48.05(39.99-57.56)	75.96(64.2-89)	291.89(242.92-349.71)	300.52(253.98-352.11)	0.09(0.07-0.12)
Western Sub-Saharan Africa	1.26(0.99-1.59)	3.28(2.58-4.16)	108.33(85.36-136.82)	140.13(110.34-177.52)	0.90(0.87-0.92)
China	14.75(11.49-18.94)	56.04(45.44-68.77)	126.78(98.77-162.78)	163.86(132.89-201.11)	0.89(0.84-0.94)

Abbreviations: ASIR: age-standardized incidence rate; CKD-T2DM: type 2 diabetes related chronic kidney disease; AAPC: annual average percentage changes.

Table S5. The age-standardized incidence rate (ASIR) of CKD-T2DM by SDI from 1990 to 2019 and the annual average percentage changes in males.

Location	15-49 years			50-74 years			≥75 years		
	ASIR_1990	ASIR_2019	AAPC	ASIR_1990	ASIR_2019	AAPC	ASIR_1990	ASIR_2019	AAPC
**SDI**									
Low SDI	2.16 (1.72-2.62)	2.39 (1.89-2.94)	0.36 (0.26-0.45)	61.32 (52.6-70.81)	78.41 (67.59-89.74)	0.85 (0.78-0.92)	111.24 (88.64-138.87)	147.75 (118.01-185.28)	0.99 (0.95-1.02)
Low-middle SDI	3.54 (2.85-4.36)	4.37 (3.51-5.38)	0.73 (0.65-0.81)	76.34 (64.87-88.37)	102.22 (88.01-116.93)	1.00 (0.91-1.09)	120.02 (93.4-154.01)	174.18 (137.04-218.72)	1.29 (1.23-1.35)
Middle SDI	3.19 (2.54-3.94)	5.84 (4.67-7.22)	2.11 (2.06-2.16)	81.69 (68.94-94.33)	114.12 (97.81-129.68)	1.17 (1.14-1.2)	157.4 (122.59-200.83)	224.39 (176.57-278.69)	1.23 (1.21-1.25)
High-middle SDI	2.96 (2.38-3.67)	4.75 (3.75-5.94)	1.65 (1.59-1.71)	77.12 (65.65-88.54)	101.32 (86.61-115.68)	0.95 (0.9-0.99)	196.29 (155.77-245.53)	252.08 (205.17-309.05)	0.86 (0.84-0.88)
High SDI	3.07 (2.36-3.88)	4.52 (3.49-5.7)	1.38 (1.19-1.58)	130.99 (111.6-150.28)	155.56 (133.8-176.51)	0.59 (0.52-0.66)	334.86 (272.92-412.77)	368.97 (308.08-442)	0.32 (0.27-0.38)

Table S6. The age-standardized incidence rate (ASIR) of CKD-T2DM by SDI from 1990 to 2019 and the annual average percentage changes in females.

Location	15-49 years			50-74 years			≥75 years		
	ASIR_1990	ASIR_2019	AAPC	ASIR_1990	ASIR_2019	AAPC	ASIR_1990	ASIR_2019	AAPC
**SDI**									
Low SDI	2.31 (1.88-2.79)	2.55 (2.05-3.09)	0.35 (0.27-0.43)	49.94 (42.18-58.6)	67.48 (57.81-78.21)	1.05 (1.00-1.09)	90.25 (70.84-114.13)	122.15 (96.73-153.53)	1.06 (1.03-1.09)
Low-middle SDI	3.16 (2.58-3.88)	3.79 (3.05-4.67)	0.63 (0.55-0.7)	62.17 (52.61-72.52)	85.76 (73.62-98.67)	1.11 (1.07-1.16)	99.02 (77.19-127.92)	140.76 (112.18-175.67)	1.22 (1.18-1.26)
Middle SDI	3.10 (2.47-3.84)	5.20 (4.15-6.43)	1.80 (1.76-1.84)	81.10 (68.45-94.54)	107.54 (93.57-122.11)	0.98 (0.93-1.02)	128.33 (99.73-164.13)	176.53 (141.63-218.02)	1.11 (1.08-1.13)
High-middle SDI	3.08 (2.48-3.77)	4.58 (3.6-5.69)	1.40 (1.35-1.45)	72.81 (61.54-84.31)	93.39 (80.12-106.38)	0.87 (0.80-0.94)	146.79 (116.7-183.13)	181.23 (148.13-219.92)	0.73 (0.69-0.77)
High SDI	3.15 (2.41-3.97)	4.28 (3.35-5.31)	1.10 (1.00-1.2)	117.51 (98.81-135.9)	130.17 (111.87-148.48)	0.34 (0.28-0.4)	281.06 (231.74-337.78)	287.52 (242.27-339.19)	0.08 (0.06-0.1)

	15 to 19	20 to 24	25 to 29	30 to 34	35 to 39	40 to 44	45 to 49	50 to 54	55 to 59	60 to 64	65 to 69	70 to 74	75 to 79	80 to 84	85 to 89	90 to 94	95 plus
2020	533	996	1977	4162	10194	23930	39617	95620	149160	205029	261388	239857	163653	94881	33353	7076	870
2021	551	1017	2002	4178	10423	24196	39323	96772	157109	209209	273028	256074	168170	97225	34317	7373	930
2022	568	1043	2023	4197	10661	24507	39162	97526	162701	215665	283923	271719	176287	99129	35453	7626	989
2023	586	1074	2049	4224	10857	24849	39085	97510	165809	227594	295050	288517	187661	101749	37022	7964	1055
2024	604	1106	2080	4267	10970	25277	39098	96770	168545	242986	304327	306315	201749	105060	38870	8373	1135
2025	623	1141	2110	4324	11030	25825	39167	95597	171408	258603	312030	324581	218342	108339	40868	8809	1221
2026	644	1178	2143	4370	11074	26432	39230	94512	173910	273497	320045	341669	235618	113126	42881	9333	1305
2027	668	1217	2184	4397	11115	27059	39288	94007	175718	284323	331911	358133	252710	120590	44731	9919	1385
2028	695	1260	2230	4428	11176	27574	39432	94193	176013	290528	351887	374001	269941	129703	46556	10524	1471
2029	725	1305	2283	4470	11277	27872	39642	95396	174895	295870	376814	386691	287465	140211	48429	11137	1564
2030	758	1354	2349	4518	11424	28046	39874	97823	172989	301450	402090	397346	305587	152401	50238	11803	1662

Table S7. The predicted global incidence cases of CKD-T2DM of different age groups in males from 2020 to 2030.

	15 to 19	20 to 24	25 to 29	30 to 34	35 to 39	40 to 44	45 to 49	50 to 54	55 to 59	60 to 64	65 to 69	70 to 74	75 to 79	80 to 84	85 to 89	90 to 94	95 plus
2020	478	828	1650	3620	9084	21531	34657	85374	138644	196685	250074	232990	173945	115680	45616	11492	1979
2021	489	837	1658	3621	9230	21572	34170	85716	145118	200077	260497	249090	177962	118387	47002	11892	2078
2022	500	851	1661	3620	9388	21655	33792	85661	149267	205501	269940	264460	185807	120201	48421	12259	2170
2023	511	868	1667	3623	9517	21770	33481	84915	150990	215681	279145	280353	197246	122098	50225	12711	2269
2024	523	885	1677	3636	9579	21966	33244	83559	152263	228772	286416	296625	211552	124538	52288	13244	2384
2025	534	905	1684	3657	9598	22275	33056	81869	153580	241798	292147	312792	228505	127092	54420	13839	2513
2026	546	926	1695	3667	9600	22646	32875	80261	154460	253784	298135	327399	246092	131399	56522	14509	2645
2027	561	949	1710	3658	9590	23050	32706	79129	154654	261740	307430	341043	263246	138863	58317	15227	2780
2028	578	973	1726	3649	9586	23373	32619	78548	153522	265256	323691	353829	280272	148588	59857	15966	2924
2029	597	1000	1748	3645	9607	23528	32600	78775	151221	267853	344109	363622	297181	160064	61408	16701	3077
2030	618	1028	1779	3643	9653	23585	32615	79967	148310	270547	364442	371422	314082	173516	62972	17474	3246

Table S8. The predicted global incidence cases of CKD-T2DM of different age groups in females from 2020 to 2030.

Table S9. The predicted global CKD-T2DM age-standardized incidence rate per 100k of different age groups in males from 2020 to 2030.

	15 to 19	20 to 24	25 to 29	30 to 34	35 to 39	40 to 44	45 to 49	50 to 54	55 to 59	60 to 64	65 to 69	70 to 74	75 to 79	80 to 84	85 to 89	90 to 94	95 plus
2020	0.17	0.32	0.64	1.49	4.09	9.96	11.27	43.25	78.28	132.29	206.35	272.00	295.36	274.35	204.00	129.94	78.06
2021	0.18	0.33	0.65	1.47	4.11	10.10	11.15	43.06	79.58	134.65	210.22	277.65	297.35	277.16	206.22	131.10	78.72
2022	0.18	0.34	0.65	1.45	4.11	10.25	11.10	42.71	80.54	137.21	213.89	283.52	300.31	279.69	208.48	132.35	79.34
2023	0.19	0.35	0.66	1.44	4.10	10.40	11.11	42.23	81.12	140.00	217.46	289.50	304.39	281.80	210.80	133.71	79.95
2024	0.19	0.36	0.68	1.43	4.06	10.53	11.19	41.67	81.37	142.97	221.10	295.57	309.59	283.51	213.20	135.17	80.60
2025	0.20	0.37	0.69	1.43	4.01	10.62	11.31	41.12	81.34	145.92	224.97	301.68	315.77	285.04	215.63	136.71	81.31
2026	0.20	0.38	0.71	1.43	3.96	10.67	11.47	40.72	81.02	148.44	229.12	307.52	322.53	287.13	217.98	138.28	82.09
2027	0.21	0.39	0.72	1.44	3.91	10.67	11.64	40.54	80.42	150.32	233.64	313.11	329.57	290.19	220.12	139.89	82.93
2028	0.21	0.41	0.74	1.45	3.86	10.63	11.81	40.61	79.58	151.52	238.59	318.59	336.79	294.38	221.96	141.56	83.84
2029	0.22	0.42	0.75	1.47	3.83	10.54	11.95	40.91	78.59	152.12	243.87	324.22	344.18	299.68	223.50	143.29	84.82
2030	0.23	0.43	0.77	1.50	3.82	10.42	12.06	41.41	77.62	152.23	249.15	330.23	351.64	305.97	224.94	145.07	85.87

Table S10. The predicted global CKD-T2DM age-standardized incidence rate per 100k of different age groups in females from 2020 to 2030.

	15 to 19	20 to 24	25 to 29	30 to 34	35 to 39	40 to 44	45 to 49	50 to 54	55 to 59	60 to 64	65 to 69	70 to 74	75 to 79	80 to 84	85 to 89	90 to 94	95 plus
2020	0.16	0.28	0.56	1.34	3.74	9.08	10.51	38.42	70.53	118.40	175.49	223.46	246.14	229.21	161.22	96.40	55.63
2021	0.17	0.29	0.56	1.32	3.74	9.12	10.32	37.98	71.32	120.13	178.13	227.21	246.97	231.18	162.67	96.89	55.71
2022	0.17	0.30	0.56	1.30	3.72	9.18	10.19	37.39	71.74	122.03	180.59	231.16	248.51	232.82	164.16	97.50	55.79
2023	0.17	0.30	0.57	1.28	3.69	9.24	10.11	36.68	71.81	124.09	182.98	235.22	250.89	234.03	165.71	98.24	55.91
2024	0.18	0.31	0.57	1.26	3.64	9.28	10.09	35.93	71.57	126.25	185.44	239.32	254.12	234.81	167.33	99.07	56.08
2025	0.18	0.31	0.58	1.25	3.59	9.30	10.11	35.18	71.09	128.28	188.09	243.38	258.10	235.37	168.98	99.99	56.32
2026	0.18	0.32	0.59	1.25	3.53	9.29	10.16	34.58	70.31	129.80	190.95	247.18	262.57	236.30	170.53	100.95	56.64
2027	0.19	0.33	0.60	1.24	3.47	9.25	10.23	34.16	69.26	130.65	194.09	250.76	267.32	237.93	171.86	101.94	57.03
2028	0.19	0.33	0.60	1.25	3.41	9.17	10.30	33.92	68.01	130.86	197.52	254.27	272.22	240.39	172.88	102.98	57.50
2029	0.19	0.34	0.61	1.26	3.36	9.06	10.34	33.87	66.65	130.54	201.12	257.91	277.20	243.69	173.60	104.07	58.03
2030	0.20	0.35	0.62	1.27	3.33	8.92	10.37	33.97	65.33	129.79	204.55	261.85	282.17	247.74	174.18	105.19	58.62

Table S11. The predicted incidence cases of Chinese CKD-T2DM of different age groups in males from 2020 to 2030.

	15 to 19	20 to 24	25 to 29	30 to 34	35 to 39	40 to 44	45 to 49	50 to 54	55 to 59	60 to 64	65 to 69	70 to 74	75 to 79	80 to 84	85 to 89	90 to 94	95 plus
2020	31	89	242	580	1205	2962	5500	17968	25417	28570	50085	43995	30291	18362	6768	1582	157
2021	32	86	232	563	1282	2888	5190	17449	30062	27137	52814	48781	31042	18897	7094	1679	173
2022	33	86	217	538	1382	2851	4961	16794	32921	27574	54399	54286	32664	19375	7442	1782	189
2023	35	87	205	512	1463	2837	4734	15836	33312	31628	54676	60352	35203	19719	7789	1923	210
2024	36	88	196	490	1487	2880	4509	14537	32955	38521	52901	66398	38494	20062	8082	2080	231
2025	39	91	187	474	1472	3008	4304	13073	32466	46785	49978	72318	42890	20467	8401	2231	253
2026	41	94	182	453	1429	3205	4081	11730	31586	55411	47615	76574	47902	21211	8785	2384	275
2027	45	97	183	425	1368	3460	3832	10648	30457	60776	48595	79212	53662	22549	9128	2540	299
2028	50	102	185	403	1304	3668	3669	9801	28777	61650	56044	79932	59977	24500	9385	2691	330
2029	55	108	188	386	1251	3737	3613	9309	26475	61173	68548	77591	66308	26992	9644	2825	364
2030	62	114	193	369	1212	3706	3643	9262	23866	60447	83507	73564	72601	30316	9945	2974	396

Table S12. The predicted incidence cases of Chinese CKD-T2DM of different age groups in females from 2020 to 2030.

	15 to 19	20 to 24	25 to 29	30 to 34	35 to 39	40 to 44	45 to 49	50 to 54	55 to 59	60 to 64	65 to 69	70 to 74	75 to 79	80 to 84	85 to 89	90 to 94	95 plus
2020	26	66	193	538	1138	2790	4811	16374	24566	28093	49501	40404	27594	18062	7908	2256	363
2021	27	64	181	514	1199	2692	4533	15686	28975	26565	51755	45316	28206	18515	8313	2401	398
2022	28	63	167	482	1278	2632	4326	14887	31536	26924	52696	50940	29528	18923	8662	2545	431
2023	29	64	155	451	1337	2596	4117	13863	31620	30838	52289	56931	31783	19169	8936	2718	471
2024	31	64	146	424	1345	2611	3906	12598	30952	37582	50028	62604	34965	19364	9151	2910	513
2025	32	65	138	402	1315	2700	3712	11243	30140	45654	46857	67750	39297	19607	9401	3103	557
2026	35	67	133	377	1258	2847	3505	10022	28922	53930	44407	71013	44258	20175	9723	3290	600
2027	38	69	132	347	1184	3041	3281	9035	27502	58799	45151	72527	49951	21265	10024	3454	644
2028	42	73	132	324	1108	3189	3136	8248	25666	59098	51920	72195	56040	23031	10232	3592	699
2029	47	77	134	306	1044	3215	3083	7757	23382	58011	63494	69271	61852	25490	10414	3712	760
2030	53	82	137	289	993	3151	3101	7638	20923	56659	77362	65084	67216	28836	10634	3855	822

Table S13. The predicted Chinese CKD-T2DM age-standardized incidence rate per 100k of different age groups in males from 2020 to 2030.

	15 to 19	20 to 24	25 to 29	30 to 34	35 to 39	40 to 44	45 to 49	50 to 54	55 to 59	60 to 64	65 to 69	70 to 74	75 to 79	80 to 84	85 to 89	90 to 94	95 plus
2020	0.07	0.17	0.38	1.08	2.53	5.00	11.32	29.96	52.45	78.23	146.46	202.80	232.24	231.94	188.58	167.34	144.70
2021	0.07	0.17	0.36	1.02	2.67	5.14	10.70	28.65	56.11	78.97	149.92	209.57	229.78	233.71	190.64	169.27	147.13
2022	0.08	0.18	0.35	0.94	2.79	5.37	10.25	27.19	58.29	81.49	151.26	216.83	229.94	234.32	192.66	171.09	149.41
2023	0.08	0.19	0.35	0.87	2.86	5.67	9.99	25.66	58.70	86.28	150.81	224.77	232.82	233.32	194.76	172.95	151.57
2024	0.09	0.20	0.35	0.80	2.85	6.02	9.91	24.08	57.69	93.24	149.71	233.35	238.30	230.49	197.02	174.98	153.70
2025	0.09	0.21	0.36	0.75	2.76	6.40	10.01	22.53	55.87	101.43	149.33	241.97	245.92	226.57	199.30	177.17	155.82
2026	0.10	0.22	0.37	0.71	2.61	6.77	10.30	21.31	53.49	108.62	150.91	247.98	254.42	224.42	201.04	179.32	157.80
2027	0.10	0.23	0.39	0.69	2.42	7.08	10.76	20.45	50.83	113.00	155.93	250.52	263.57	224.87	201.83	181.45	159.70
2028	0.11	0.24	0.40	0.69	2.23	7.26	11.38	19.96	48.04	113.95	165.35	250.15	273.62	228.02	201.27	183.70	161.68
2029	0.12	0.25	0.42	0.69	2.06	7.25	12.10	19.82	45.16	112.18	178.98	248.74	284.54	233.78	199.15	186.14	163.84
2030	0.14	0.27	0.44	0.71	1.93	7.03	12.89	20.06	42.33	108.84	195.07	248.55	295.60	241.70	196.13	188.64	166.20

Table S14. The predicted Chinese CKD-T2DM age-standardized incidence rate per 100k of different age groups in females from 2020 to 2030.

	15 to 19	20 to 24	25 to 29	30 to 34	35 to 39	40 to 44	45 to 49	50 to 54	55 to 59	60 to 64	65 to 69	70 to 74	75 to 79	80 to 84	85 to 89	90 to 94	95 plus
2020	0.07	0.14	0.33	1.06	2.50	4.86	11.49	27.63	50.28	73.69	130.52	163.61	174.94	166.67	129.29	102.18	77.85
2021	0.07	0.15	0.31	0.99	2.62	4.95	10.80	26.10	53.62	74.14	132.73	168.54	171.77	167.29	130.35	103.33	79.01
2022	0.08	0.15	0.30	0.91	2.71	5.13	10.29	24.47	55.36	76.48	132.84	173.86	170.86	166.88	131.37	104.30	80.09
2023	0.08	0.16	0.29	0.82	2.75	5.37	9.96	22.85	55.28	81.11	131.33	179.69	172.24	165.10	132.44	105.18	81.15
2024	0.08	0.16	0.29	0.75	2.72	5.67	9.80	21.27	53.83	87.85	129.36	185.96	175.72	161.86	133.61	106.11	82.27
2025	0.09	0.17	0.30	0.69	2.61	5.98	9.81	19.79	51.62	95.64	128.26	191.96	180.81	157.80	134.78	107.15	83.45
2026	0.10	0.18	0.31	0.65	2.44	6.27	10.01	18.62	48.84	102.14	129.23	195.50	186.54	155.18	135.48	108.18	84.52
2027	0.10	0.19	0.32	0.62	2.23	6.50	10.38	17.78	45.87	105.63	133.54	196.01	192.76	154.63	135.38	109.22	85.47
2028	0.11	0.20	0.33	0.61	2.03	6.61	10.90	17.24	42.91	105.69	141.91	194.17	199.63	156.19	134.21	110.33	86.36
2029	0.12	0.21	0.35	0.61	1.85	6.55	11.52	17.00	40.04	103.16	154.04	191.68	207.05	159.69	131.87	111.55	87.31
2030	0.14	0.23	0.36	0.63	1.71	6.29	12.18	17.07	37.34	99.16	168.11	190.52	214.26	164.73	128.88	112.81	88.39

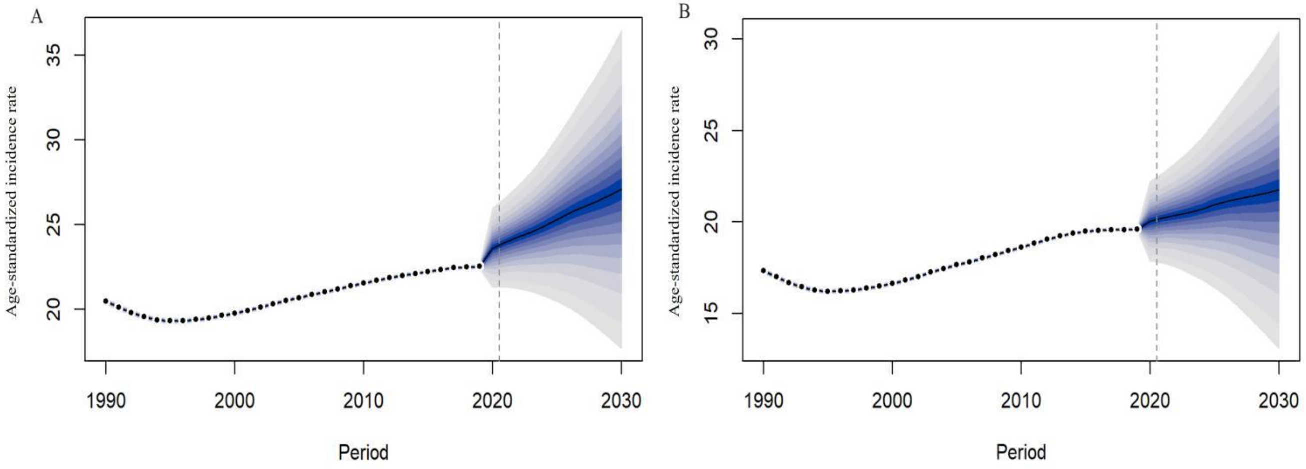

Figure S1. The prediction of Chinese CKD-T2DM incidence rate from 2020 to 2030. (A. Males, B. Females).

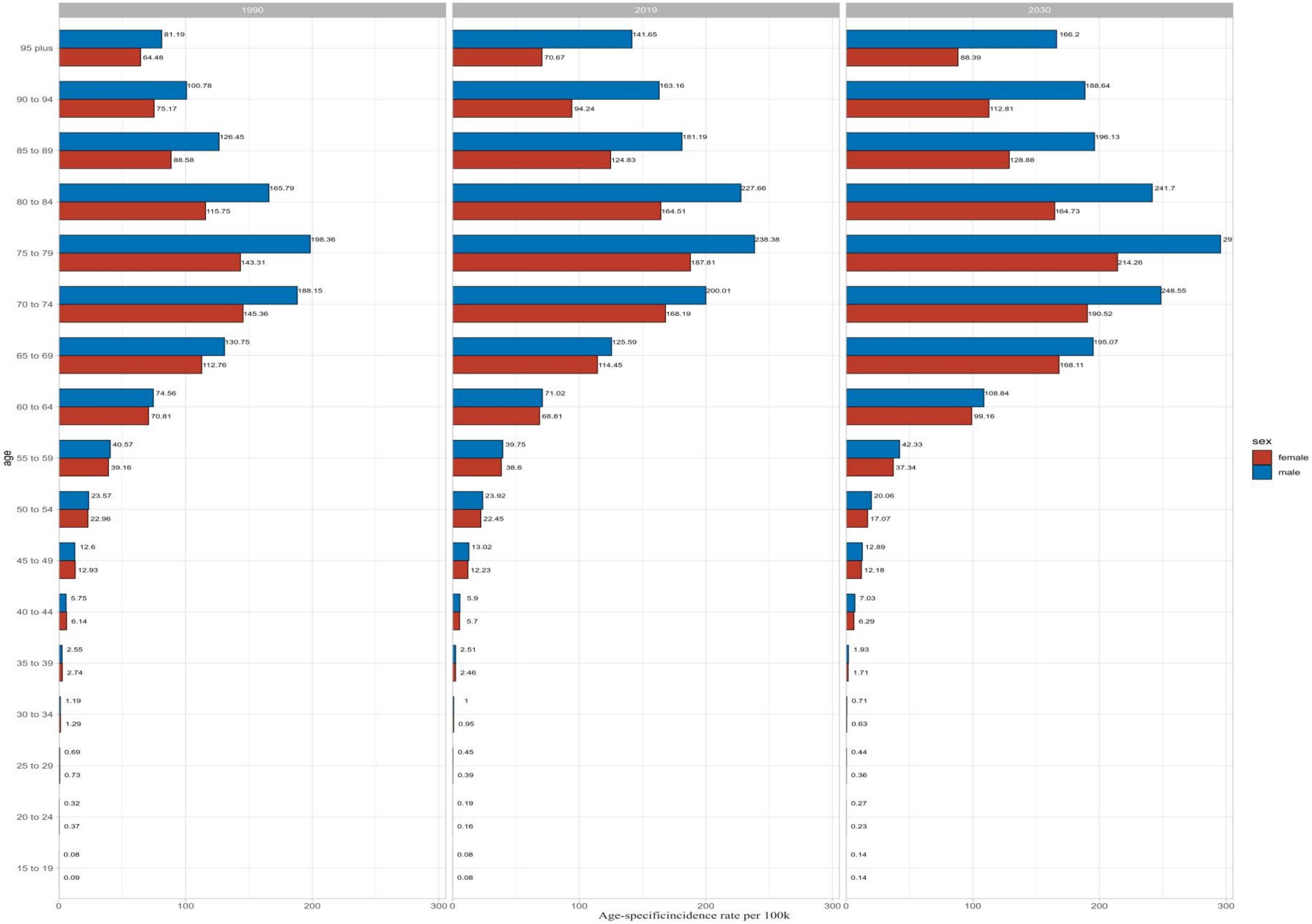

Figure S2. The prediction of Chinese CKD-T2DM incidence rate by age in 1990, 2019 and 2030.
